# GRANDPARENTS CARING FOR GRANDCHILDREN: HOW AGE AND AGING IMPACT PROVISION OF CHILDCARE

**DOI:** 10.1093/geroni/igz038.2649

**Published:** 2019-11-08

**Authors:** MaryBeth A Apriceno, Stacey B Scott, Sheri Levy

**Affiliations:** Stony Brook University, Stony Brook, New York, United States

## Abstract

The economic need for dual-income households has contributed to more grandparents providing childcare for their grandchildren. Research on these grandparents has examined their life satisfaction, health, and spare time. Little work to date has examined how cross-sectional differences in grandparents’ age may contribute to when they begin providing childcare or how their increasing age while caregiving influences when they reduce or stop providing childcare. Using Health and Retirement Study data, we identified 5.38% of participants (N=516) who reported providing at least one hour of childcare for their grandchildren per wave (range=1-9,996) between 2004 and 2014. The resulting sample ranged from 44-88 years of age (M=59.78, SD=7.75) when they first reported providing childcare; 48.8% were retired during the study period. Using multilevel modeling, we tested age and retirement as predictors of individual differences in initial amount of childcare (intercept) and change in childcare (slope). Cross-sectionally, no significant differences in childcare hours were observed based on the age at which grandparents began caregiving or retirement status. Longitudinally, however, as grandparents got older during the study, hours of childcare decreased by 48 hours per year (B=-48.368, t(1584)=-6.55, p<.0001) relative to when they starting providing childcare. These results suggest grandparent childcare providers represent an age diverse group managing multiple roles and responsibilities who continue caregiving well into older adulthood. Future research on grandparents who provide childcare for their grandchildren should observe these individuals over time to better understand how aging impacts provision of care and to examine how aging may moderate previous cross-sectional findings.

